# What qualities are required for globally-relevant health service managers? An exploratory analysis of health systems internationally

**DOI:** 10.1186/s12992-019-0452-3

**Published:** 2019-02-06

**Authors:** Reema Harrison, Lois Meyer, Ashfaq Chauhan, Maria Agaliotis

**Affiliations:** School of Public Health and Community Medicine, University of New South Wales, Sydney, NSW 2052 Australia

**Keywords:** Health leadership, Global health, Health services, Health service management

## Abstract

**Background:**

Globally, health service leaders and managers have a critical role in strengthening health systems. Competency frameworks for health service managers are usually designed to describe expectations of good performance of a health manager within a country-specific health sector context. However, a growing number of health service management roles operate beyond a country-specific level, with managers requiring a global perspective and the skills and knowledge to work effectively across a range of countries and contexts. This study provides an exploratory analysis of the most relevant qualities perceived to facilitate health service managers to be effective when working in such roles.

**Methods:**

A qualitative, descriptive, and exploratory multi-centre study was undertaken. Semi-structured individual interviews were conducted between August and November 2017 with 15 health service managers or leaders at a range of levels from seven countries [Australia (5), China (2), Indonesia (3), Oman (1), Papua New Guinea (1), United Kingdom (1) and United States of America (2)].

**Results:**

Five themes emerged relating to the qualities required from health leaders in order to be effective when working in a global context: i) Managing and Making Change, ii) Collaborative Managers and Compassionate Leaders, iii) Continuous Learning, iv) Balancing Management Theory and Practice, and v) Leadership Skills.

**Conclusion:**

Our findings highlight underpinning themes relating to making and managing change and collaboration, reflecting the changing needs of health services to deliver high quality care. These themes were identified in addition to some of the common qualities required of healthcare leaders and managers that are identified that reflect many country-specific frameworks.

## Introduction

The World Health Organization’s (WHO) landmark publication ‘Working together for health’ highlighted not only the global health workforce shortage, but also the critical role of health leadership and management as fundamental in strengthening health systems globally [1]. The recent formation of the Global Consortium for Healthcare Management Professionalization has sought to raise the recognition of professional management in healthcare, and in particular to identify the health management capacities required to meet the current and emerging global health workforce. The Consortium has made an urgent call for a global and context sensitive perspective to understanding and fostering the development of health service managers [2]. Yet to date how this might best be understood and reflected within the realities of health management practices across diverse health sector settings has not been well investigated.

Globalisation, and the increasing flow of people, products and services in the health sector between countries is impacting health service delivery [3, 4]. A growing group of health service leaders and managers now work across more than one setting, either concurrently or throughout their career. The boundaries between different health systems are becoming indistinct and healthcare managers are often required to work as boundary riders [4, 5]. For example, those who lead or manage health programs or services operated by multi-national providers or non-government organisations (NGOs) (eg. World Health Organization, Bill and Melinda Gates Foundation, Save the Children) are typically working in international settings often having highly mobile careers responding to shifting global health needs and emergencies. Furthermore, mobilisation of health professionals shifting from their home country to seek better educational opportunities and careers who then become managers creates clinician managers with knowledge of their own health system as well as crossing into new health service spaces. The expatriate health management workforce brings both potential opportunities and challenges [6, 7]. Those who have worked in multiple locations may have a broader knowledge of disease types, treatment options, service delivery models and demonstrate cultural competence while also needing to adjust to the multiple demands of professional practice in a new context [4, 8]. More recently, the need for a particular new competence that addresses the complexities of boundary crossing in health settings and nations has been called upon for those working at a global level: transnational competency [9].

In an attempt to develop internationally relevant competencies that could be applied within country specific contexts, the International Hospital Federation (IHF), with its partners, have adopted a directory of core healthcare management competencies derived from Health Leadership Alliance [10, 11]. The core competencies are those that are commonly identified across a range of settings internationally: 1) leadership, 2) communication and relationship management, 3) professional and social responsibility, 4) knowledge of health and healthcare environment, and 5) business [10]. These competencies are argued to be critical for health managers and leaders in different health service sectors. Although the competencies mentioned above are supported by WHO for strengthening health leadership across systems, the capabilities required of health managers and leaders for boundary crossing in their own careers and addressing complex health issues across various health systems are less well understood.

Current approaches in understanding global health management do not fully attend to the needs of the above groups of health managers that are required to adopt a global perspective. The current qualities required from health service leaders and managers across different health service settings globally to ensure they can manage their workforce, improve health outcomes and work within the increasingly interdependent global health context are not yet well understood. This exploratory study aimed to provide insight regarding the qualities that are particularly important to facilitate effective practice as a health manager or leader when working across multiple countries.

## Methods

### Ethical approval

Ethical approval was granted by the University of New South Wales’ Human Research Ethics Committee (HC17270).

### Design

A qualitative, descriptive and exploratory multi-site study was conducted between August and December 2017.

### Sample

The sample was purposively selected to capture views of key informants who were health service managers who had experience of more than one health system, from a range of levels and from a range of countries across the globe. Given the diverse range of systems, services and locations from which health service managers could be identified, we did not seek to achieve saturation through our sampling approach. Rather, we sought to identify a diverse group of managers working in different geographic locations and levels to address the research questions posed. Email invitations were initially sent to 20 potential participants who were identified through professional links with the University of New South Wales’ Health Management Program, which is a large and well-established postgraduate program.

### Procedure

Semi-structured interviews allowed in depth data to be gathered with the flexibility to pursue interesting lines of enquiry. Face to face or telephone audiotaped interviews lasted between 30 and 50 min. The interview schedule included three core topics exploring: 1) key features of global healthcare practice; 2) challenges facing healthcare managers and leaders globally; 3) the critical qualities required of healthcare leaders and managers to work at a global level. The schedule was reviewed and refined following pilot work with health service managers in Australia. Interviews were conducted incrementally, and thematic content analysis was conducted concurrently in an iterative process to address the research questions posed [12, 13]. Whilst we did not try to achieve data saturation, we applied an iterative approach to data collection and analysis that enabled us to continually assess if data saturation was reached.

### Analytic strategy

Thematic analysis allows a theoretically-flexible approach for identifying, analysing and reporting patterns in qualitative data and is useful for obtaining detailed insights into complex issues [14]. The interviews were transcribed verbatim and managed used NVivo (version 11). Transcripts were independently read by two researchers (RH, AC) who, once familiar with the breadth and depth of content, undertook a focused line by line analysis. Themes were generated from the initial coding and then grouped under broader categories that were then labelled with reference to the raw data. A third researcher (MA) read a subset of five transcripts to validate the themes and categories proposed. Different interpretations were resolved through discussion in this process. This investigator triangulation method further assisted to confirm the observations, add to the breadth of the analysis and help validate the results of the study [15, 16]. Respondent validation was conducted to help improve the accuracy, credibility, validity, and transferability of the findings through presentation and discussion of the preliminary findings to a sub-sample of the respondents on an individual basis.

## Results

The sample comprised of 15 participants from seven different countries; nine from high-income countries and six from low- or middle-income countries [Australia (5), China (2), Indonesia (3), Oman (1), Papua New Guinea (1), United Kingdom (1) and United States of America (2)]. Participants worked as junior level health managers (6); middle (3) and senior health managers (4) or C-Suite executives (top level management roles such as Chief Executive) (2) across government (7), private for profit (5) and not for profit (3) sectors. This approach facilitated capture of perspectives across a diversity of geographical and health care settings.

Five themes emerged relating to the qualities required of health leaders to be effective when working in a global context: i) Managing and Making Change, ii) Collaborative Managers and Compassionate Leaders, iii) Continuous Learning, iv) Balancing Management Theory and Practice, and v) Leadership Skills.

### Managing and making change

The majority of the participants described healthcare as in a state of constant change and identified a need for health managers and leaders to be ready to manage change for delivering effective and efficient healthcare. Factors driving change such as the availability of resources, organisational restructure, technological advancement and changing patient expectations were consistently noted.

P7 (HIC) *- “Change management is a reality for health care managers.”*

P13 (HIC) *- “I think change management skills are absolutely crucial in the health space. It’s a space which is in a constant state of flux and we don’t do change well and that’s part of the reason why we’re in a constant state of flux, is because we do things poorly and then have to fix them afterwards, or deal with unintended consequences.”*

P1 (HIC) *- “The major challenges are one, being able to implement change. So, change - yeah, implementation.”*

The importance of not only being able to manage but also to create change as a manager was further identified by some senior and middle level managers. Respondents described the need to adopt innovative approaches, think in advance and flexibly in relation to their change management strategies. With an increasing pace of change, health leaders need to be creative and innovative rather than perceiving change as disruption. By creating leaders and change managers that can think innovatively and with an open mind is important to address the fast pace of change in healthcare.

P8 (HIC) - *“I believe that in my experience it has been far more useful being good at creating change than being good at reacting to it and that’s the bit that I don’t think we’re doing as well as we could.”*

P8 (HIC) - *“I don’t think there’s been a lot of re-imagination of what we could do, how we could do it, what we’re doing, what needs to change and how should we be preparing the next generations of managers to be able to do all of the things that we hope that they might be able to do.”*

P6 (LMIC) *- “The most important is to have an open mind and to have - can do a lot of change, because the healthcare environment changes very fast, and we need to follow up to the speed and just change yourself and also to accept an open mind, to be an open mind to encourage the creative thinking in your team.”*

A key component of effective change management was identified as an ability to consider impacts on staff and patients and to proactively address these to gain buy-in.

P11 (HIC) - *“I think when you look at any change, you need to look at the impact on the patients and the quality of the services that you deliver… if we can get a step ahead and really understand how it impacts on our staff, that helps us change better.”*

P11 (HIC) – *“If you can get that social movement and hearts and minds and focus the patient in the centre of it, that’s what actually turns clinicians on, because they are patient focused. That makes change all that bit more easy”.*

P1 (HIC) *- “I think that they’ll need to have a good understanding of how to go about behaviour change, winning over hearts and minds.”*

### Collaborative managers and compassionate leaders

Whilst competencies such as being able to ‘communicate’, ‘have a strategic vision’, ‘be a good problem solver’ and ‘motivate others’ were highlighted by most, the importance of a health manager or leader to be able to collaborate with a range of stakeholders both internally and externally was identified as a key underpinning quality. Collaborative skills were discussed in the context of developing effective multi-disciplinary teams, capacity building, patient involvement and managing service delivery to address an increasingly complex range of health conditions.

P10 (HIC) *- “I think there’s a bigger awareness around the need of collaboration. The need of everybody knowing what everyone else is doing in the system and then figuring out more attractive ways to work together on our problems.”*

P10 (HIC) - *“I think it’s important again to be interdisciplinary and then to be able to work effectively with a lot of stakeholders. I think that’s a mentality that has to shift for the healthcare system in order to be effective.”*

P1 (HIC) - *“Bringing people along with a - if you like, a co-developed, co-produced vision and making it happen.”*

In terms of patient and family engagement, participants noted the importance of seeking patient and family perspectives and contribution, but also relating to them with compassion. Senior health executives within the group highlighted attributes such as honesty and authenticity as critical within this process. Respondents converged on the notion of being able to go beyond the role of a leader in a clinical setting and understand the moral obligation they have towards patient-centred care.

P15 (HIC) *- “We’ve seen a great trend towards understanding from the patients’ view rather than, I’m doing it and the patient needs to just abide by what I write as a care plan and that’s it.”*

P9 (HIC) *- “We just want to train compassionate leaders that, even though they’re not directly interacting with the patient they understand that they have to have the ability to really improve the way that patients are treated.”*

P11 (HIC) - *“I think we should be open, we should be honest. We should genuinely take an interest in our staff and our patients. We should be able to focus everything we’re doing around the purpose of why we’re here, which is for the good and the benefit of the patients. I think if you can then create a team around you that is authentic, and role model similar behaviours, that then cascades down throughout an organisation. I think it’s about hearts and minds, values, being an authentic, visible leader but with honesty and integrity as well.”*

Collaboration was discussed in the context of capacity building by those from middle-income countries. For example, participants from China discussed how their hospital, by building collaborative learning opportunities with teaching institutes has enhanced the capacity, skills and knowledge of their health managers and leaders. These respondents noted that a collaborative learning effort must be underpinned by an organisational culture of learning and desire to develop skills and capabilities of their staff.

P2 (LMIC) *- “Globally institutions that are willing to do the collaborations, obviously they have seen these advantages by doing these. Often, we say one plus one actually bigger than two. So that’s the reason these steps will going on in the future.”*

P6 (LMIC) *- “So just this team, interns visit here [Australia],[and when] they come back to China. They just build several programmes by themselves to develop not only for themselves to keep learning but also for their staff.”*

### Continuous learning

The need to engage in continuous learning as a health service manager was widely recognised by all participants, but the ability to do so was identified as an important skill in itself. Respondents cross all the interviews from different countries and at all levels from junior managers indicated that health leaders and managers should be proactively seeking opportunities to improve their knowledge.

P10 (HIC) *- “Once you come out of school you still have to learn and continue learning. I think if you’re an effective learner you will succeed. If you are less willing to learn you will not succeed. You have to always keep learning and that’s also a very essential skill for any leader. It’s the ability to learn and to keep learning because things will always change, and you have to continue learning new things”.*

*P6 (LMIC) - “Keep learning - keep learning is very important, yes.”*

The opportunity to expand one’s knowledge was identified as a key gain to be made from international and cross-organisational collaboration. Respondents described keeping an open mind and being able to learn from others as an essential capability in order to do so effectively. Globalisation of health care delivery brings with it opportunities for the managers and leaders to learn from different healthcare systems.

P11 (HIC) *- “I think it’s absolutely essential...to keep an open mind and draw on the examples from other countries. We are so well connected as a planet now, why would we just choose to speak to our local hospital or neighbours on what was good practice, or how to make improvements?”*

P15 (HIC) *- “So health care is global. I believe that we as health managers need to be globally knowledgeable in what is going on in health care systems everywhere.”*

P13 (HIC) *- “I think there’s a huge amount of value. You can’t be too blinkered in the way that you do things. I think you’ve always got to maintain a little bit of that - almost an epistemological humility and be willing to accept that the way that you’re used to doing things isn’t necessarily the best way.”*

Two participants described how when spending time in other healthcare systems, health managers and leaders must not only focus on the outcomes achieved, but also learn from their journey and develop a reflective and analytical skills. One participant suggested that it is imperative that the leaders first understand that their healthcare system is not perfect in order to reflect on their own system and others.

P13 (HIC) *- “Having some focus on what things are like in different contexts and gives you an opportunity to be able to reflect on whether there might be better ways of structuring the way that you allocate resources or organise your healthcare system.”*

P15 (HIC) *- “We also need to be flexible enough to take from what works in another country and look at it in a way that we could implement it here or start - it’s not always sort of a cut and paste that works - it’s about how did they reach there.”*

### Balancing management theory and practice

Almost all recent graduates identified transitioning from a recent graduate to a healthcare manager as a difficult process. Senior managers and executives also identified that junior managers or recent graduates lack experience related to application of theory into practice. But, they also identified that health managers also need theoretical knowledge for evidence-based practice. Alternatively, almost all recent graduates also mentioned that they use their theoretical knowledge related to health management training to develop evidence-based practice at work.

P2 (LMIC) - *“Junior people just graduated from the universities, actually they lack half the experience. I mean, that’s not just the thing only happen in Shanghai or China, that’s actually happen all over the world.”*

P13 (HIC) *- “It was all about applying the learning to your workplace.”*

P3 (LMIC) *- “I’m using the knowledge and understandings from the courses in practice…. The courses have given me the foundation.”*

Almost all participants agree that some form of practical experience at the university level will help develop essential practical skills among the junior managers. In addition to this, few participants also mentioned that it is important for health organisations and senior managers to create opportunities for junior managers to develop practical skills.

P10 (HIC) *- “I know the program does help but I think being on the ground and then working with people I think shapes you a lot. I think you become more effective working on several projects and figuring out how to deliver those expectations on time, on budget and ensuring things happen quickly.”*

P8 (HIC) *- “I guess the discussion we’re having is similar to the discussion about basic research and translational research. So it’s all very well to teach people what you can do in a test-tube. We now know that a lot of things that make a lot of sense in a test-tube are either not appropriate outside that test-tube or just can’t be implemented and I think we have to talk about that implementation science far more than we have been.”*

### Leadership skills

In addition to practical knowledge about running the programs on budget and completing within their timeframes, one participant also mentioned that it is important for leaders to develop skills for emotional intelligence. It was highlighted that the practical skills of a leader or a health manager requires more than translation skills. An effective leader should be able to control their emotions and show resilience and courage and be sensitive to the context, culture and the circumstances around them. While another participant also mentioned that it is important for health leaders to be able to listen to others and take feedback from them. This was mentioned as an important attribute in a leader in a constantly changing healthcare environment.

P7 (HIC) - *“You may know the facts and know what to do and how to do it but giving people practical skills and reflection, so the emotional intelligence component of the skillset is really, really important…. think we make the mistake of making sure people have all of the academic requirements covered. But perhaps not the resilience and emotional intelligence training to be able to fulfil the role.”*

P14 (LMIC) *- “For me skills is, of course, very important but it’s the application of those skills and I think the application comes with being sensitive to your circumstances and your context. That includes culture, it includes leadership as well.”*

P10 (HIC) *- “I think if you’re able to effectively listen and take feedback from individuals you can really use a lot of that information to make changes that are more effective and well hopefully change the way healthcare is delivered for the positive.”*

## Discussion

Our findings revealed five themes that illuminate core qualities required of health managers working globally: i) Managing and Making Change, ii) Collaborative Managers and Compassionate Leaders, iii) Continuous Learning, iv) Balancing Management Theory and Practice, and v) Leadership Skills. Themes relating to making and managing change and to collaboration were particularly pronounced in the data, underpinning the other themes and reflecting the changing needs of health services to deliver high quality care [17].

Healthcare services internationally are experiencing continual and increasingly rapid change with the proliferation of innovative treatments and approaches along with catering to dynamic and aging populations [17]. As such, our data reflects the critical need for contemporary health service leaders and managers to have the ability to respond and adapt to changes, but to also use the context of continual change to advantage to spearhead change that will enhance service delivery.

An ability to collaborate not only across health care teams but also with patients, other services and other sectors was identified as important for health leaders to be effective at a global level. This reflects an increasingly integrated, multi-disciplinary approach in contemporary health care delivery engaging a range of stakeholders [18]. Central to this also, is achieving the patient-centred care agenda; substantial emphasis has been placed on collaborating with patients to co-create models of service delivery [17]. Patient-centred care strategies are evident across a multitude of health services internationally [19].

Respondents also identified collaboration in the context of human resource capacity building, particularly in low and middle-income countries. Collaborative approaches for capacity building are identified in the WHO Human Resources for Health Framework as a strategy to achieve human resourcing goals in health services internationally [20]. Collaboration across sectors and services may provide health services in LMICs with a range of potential benefits such as leveraging resources across multiple sites or countries, reducing the risk of taking up a new initiative or providing access to complement or develop existing expertise [20].

### Implications

When considering the data against current major competency frameworks, our data reflect common features central to leadership and management such as continuous learning, compassionate leadership and leadership skills to inspire, manage and hold individuals, teams and oneself to account [2, 11, 21]. Major competency frameworks largely originate from western health systems and relate to the leadership and management goals within these. In pooling leadership and management competencies through an international Task Force, The Health Alliance Competency Directory provides a broader set of competencies that more closely reflect our data e.g. ‘Communication and Relationship Management’ linking to internal and external individuals and groups [22]. Change making and management along with collaborative approaches however do not feature explicitly in any of the above frameworks. Our findings indicate that these qualities are central to the ability of health leaders and managers to work effectively in a global context and may warrant consideration in competency frameworks.

The concept of transnational competence; the requirement for health care workers and managers to hold ‘analytic, emotional, creative, communicative, and functional skills’ in order to provide effective and appropriate migrant healthcare, is also relevant to our work [9]. Koehn and Rosenau argue that notion of cultural competence, included in current competency frameworks, does not sufficiently extend to the multiplicity of cultures that health care workers, leaders and managers are exposed to in contemporary healthcare. To date, transnational competence has been explored largely in the clinician-patent dyad rather than in the context of health service management. Our findings provide an initial indication that additional analytic, emotional and communicative skills are required of healthcare managers and leaders to work transnationally, but further work to understand the nature of transnational competence as applied to health service management and leadership would be valuable.

Our findings also have implications for postgraduate education in health management. Our data emphasise the need for health leaders and managers to be responsive to change, but also to use collaborative links and networks to bring about change. Postgraduate health management education generally provides context-specific education relating to discrete management competencies identified in the relevant competency framework/s [23]. Such approaches provide knowledge and skills to manage workforce, budgets, and to monitor the quality of care delivery in any given health system. Work integrated learning across health systems may be one approach to enable students to build a portfolio of evidence demonstrating leadership and health service management capabilities in a range of contexts in order to generate health leaders and managers with the relevant skills and knowledge to work globally.

### Limitations

As an exploratory study although participants were drawn from a range of locations and health services, there are many more perspectives of health service managers and leaders across the globe whose perspectives may not have been recognised here. Health service leaders and managers working in other parts of the sector or locations may have contributed different experiences or perspectives regarding the needs of health managers in a global context. Those working in health services at a senior level may also be inhibited in their responses due to their role and position in their organisation.

## Conclusion

Whilst common qualities of healthcare leaders and managers were established that reflect many country-specific frameworks, the data highlights the ability to make and manage continuous change and to collaborate effectively (both internally and externally) as key qualities specifically valuable for global health management roles. These qualities align with contemporary models of health service delivery and reflect the impact of globalisation on health care provision, with implications for competency models and postgraduate education of the health management workforce.
